# Decelerating Mortality Rates in Older Ages and its Prospects through Lee-Carter Approach

**DOI:** 10.1371/journal.pone.0050941

**Published:** 2012-12-06

**Authors:** Awdhesh Yadav, Suryakant Yadav, Ranjana Kesarwani

**Affiliations:** International Institute for Population Sciences (IIPS), Mumbai, India; Sapienza University of Rome, Italy

## Abstract

The present study attempts to study the age pattern mortality and prospects through Lee-Carter approach. The objectives of the study are to examine the trend of mortality decline and life expectancy. Contemporaneously, we have projected life expectancy up to 2025, projecting ASDR using Lee-Carter method. Life table aging rate (LAR) used to estimate the rate of mortality deceleration. Overtime, LAR increased and during recent decade it remained more or less unchanged. By age, LAR significant increased in the oldest of old. The slope is steepest in the oldest of old in the recent decade. The rates of mortality increased in oldest of old as the age group is more vulnerable to chronic disease and vulnerable to identifiable risk factors for virtually every disease, marked by senility. The analysis revealed that the level of mortality is not declining but rate of acceleration is declining and is further expected to decline. By the year 2025, the age specific death rates for the age group 5–9 and 10–14 will go below one per thousand.Life expectancy will attained as high as 73 and 79 years for male and female and is further expected to increase linearly. 71 percent of total female birth and 57 percent of total male birth will survive up to age 70+. Also the findings revealed that mortality rate is declining with constant rate up to age 70 and thereafter, the mortality rate accelerates and this holds true for both sexes.

## Introduction

Kingsley Davis spoke of the “amazing decline” of mortality in underdeveloped countries [1]. According to WHO report, India rank third, next to only Myanmar and Nepal, among all South Asian countries ordered by adult death rate in 2001 [2]. One of the greatest human achievements has been the decline in mortality that has occurred during the modern era. Substantial mortality decline in other part of the world is a more recent phenomenon, sharply accelerating after 1950; although demographic data to document these trends are deficient in most of the all part of the third world countries. It is shown that mortality decline in India might have been due, speaking, to development rather than as a primary consequences of public health.

The age pattern of mortality has drawn considerable attention in the field of applied demography and public health not only because mortality is one of the direct determinants of population change but also because of the risk of mortality. Also, the cause of death would be very different in various age segments and any health policy therefore has to take this into consideration the age pattern of mortality. It is generally noted that children in infancy and aged people are most vulnerable group. The study of age pattern is very important from the point of view of planning. While projecting the population, the future age pattern of mortality is borrowed from sets of model life tables (MLT). Therefore the present study tries to model the pattern and prospects of mortality in older age in India.

Literatures suggest different models for forecasting mortality not all are suitable in Indian conditions. For instant, the 8- parameter model developed by Heligman and Pollard (1980) [3] has been found to fit a wide range of mortality schedules, but the large number of parameter limits the model’s potential for projection particularly in the context of developing countries [4]. Lee and Carter (1992) [5] proposed a simple method for forecasting future mortality, consisting of a base model of age specific death rates with a dominant time component and a fixed relative age component, and a time series model (autoregressive integrated moving average (ARIMA)) of the time component. A major problem with the Lee carter method is the assumption that the age component is invariant over time [6].

In a study of mortality in India and all the major states as well as, Roy and Lahiri (1987) [7] showed that the age pattern of mortality in India, for males and especially females, resembled well the South Asian Pattern of the new U.N. model life tables (1982) [8] and also the South pattern of the Coale- Demeny model life tables [9]. To study the trends in mortality, life table for each year’s during the period from 1970 to 1983 was constructed and the indices like _5_L_0_/l_0_, _10_L_0_/l_15_, _30_L_15_/l_15_, and _∞_L_50_/l_50_ were used to study the mortality at age 0–4, 5–11, 15–49, and 50 over. In addition to these indices, the e_o_° and q_0_ was also used. The mortality in India was found to be higher at both ends of life span. Female mortality was higher in comparison to male mortality, except at the older ages. The improvement in female mortality was much faster than the male mortality.

To get the average rate of decline in the death rates at each five years age intervals, Bhat and Navaneetham (1991) [10] regressed the logarithm of death rates from the sample registration system on time. The analysis showed that mortality declined more rapidly among the infants, children below 15 years of age and women in the reproductive ages. Among adults, mortality declined more rapidly among females than in males, the differential being largest in the age group 25–49.

**Figure 1:**
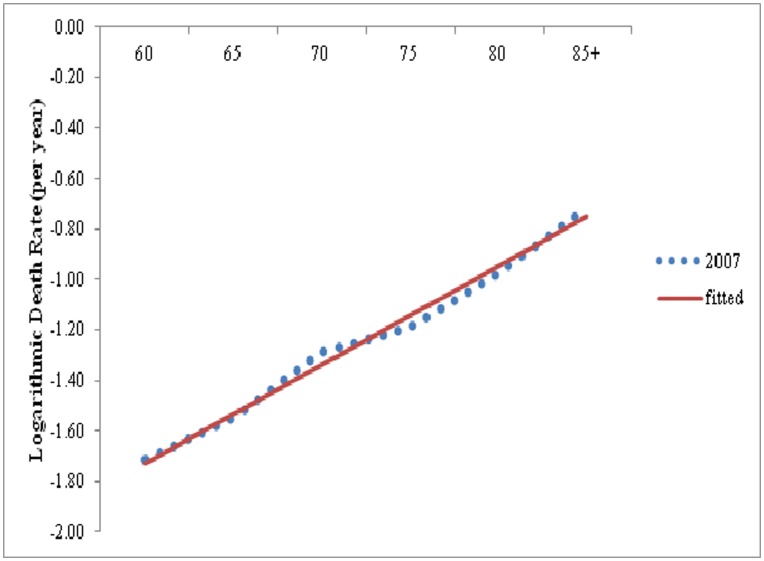
Age Specific death rates for female on logarithmic scale, India, 2007 (*Note*: Dotted are logarithmic of death rates between 60 and 85+. The line was fitted to death rates between age 60 and 85 by ordinary least squire regression).

The study of age pattern of mortality in older age is essential for most of demographic measurement and forecasts. In most of the indirect technique of fertility estimation, one needs the knowledge of age pattern of mortality as well as the level of mortality. It has been supposed that human mortality from all cause increases with age nearly exponentially (at constant rate) through adult age except for very older ages, and that this exponential increase also holds fairly well for most major cause of death (CODs) [11]. When age specific death rates are plotted against age on a logarithmic scale, points appear to fall along a straight line as illustrated for Indian females in **figure 1**. Thus the present study make an attempt to study the life expectancy in future and the scenario of oldest old mortality i.e. whether mortality is accelerating or decelerating in the older age groups.

## Methods and Materials

Sample Registration System (SRS) [12] is only source that provide age specific death rates, sex and place of residence (Rural, Urban and Total) for all the major states of India. SRS collects information based on the concept of dual record system and thus, errors due to sampling and non-sampling errors are expected to be less. Bhat (2000) [13] pointed out the incompleteness in death-registration in the SRS death records which are approximately 0.2 percent; in the context paucity of other sources of data and their relative reliability compared to the SRS data, it is worthwhile to use information available with SRS. The present study has made use of age specific death rates by sex from 1981 to 2006 for India. The abridge life table based on SRS data starting from 1978 for India has also been used to observe the trend of life expectancy [14].

The one way to examine the age variation in mortality is to plot the logarithm of the age specific death rate against age. Significant pattern of mortality acceleration or deceleration can be easily visualized through the inspection of plot between logarithmic age specific death rates and age group. A simple measure, the life-table aging rate (LAR), has proven to be a powerful tool for detecting these patterns [15]–[20]. The LAR at exact age x is defined as:
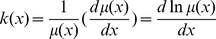



Where 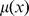 is the force of mortality (instantaneous death rate) at age x for any population. Thus, the LAR measures the relative mortality increase with age: For example, an LAR of 0.05 means that the death rate is rising (at exact age) at an exponential rate of 5% per year of age.

When data are tabulated by five-year age groups, the LAR can be estimated by
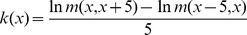



Where M(x, x+5) is the death rate for the interval between exact ages x and x+5.

For the second objective, Lee-Carter (1992) method is used for long-run forecasts of the level and age pattern of mortality, based on a combination of statistical time series methods and a simple approach to deal with the age distribution of mortality. The method describes the log of a time series of age specific death rates as the sum of an age-specific component that is independent of time and another component that is the product of a time-varying parameter reflecting the general level of mortality, and an age specific component that represents how rapidly or slowly mortality at each age varies when the general level of mortality changes. The forecasting of the index of level of mortality gas been done by Autoregressive Integrated Moving Average Model (ARIMA).

### Lee- Carter Model Description and Fitting of the Model

The model is specified as
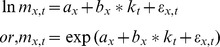
where m(x,t) is the death rate for age x in year t, the a_x_ coefficient describe the average shape of the age of the mortality, the b_x_ coefficient describe the pattern of deviations from this age profile when the parameter k varies and k_t_ represent the index of level of mortality. As k goes to negative infinity, each age-specific rate tends to be zero and hence negative death rates cannot occur in this model, which is an advantage for forecasting.

The equation of the model is
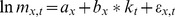



The estimates of a_x_, b_x_, and k_t_ can be obtained by normalizing b_x_ to sum to unity and the k_t_ to sum to zero. The a_x_ values can be estimated by averaging ln (m_x,t_) over time. Further, k_t_ can be estimated by summing over the age of [ln (m_x,t_) –a_x_]. Also, b_x_ can be estimated by regressing, without a constant term, [ln (m_x,t_) –a_x_] on k_t_ separately for each age group x.
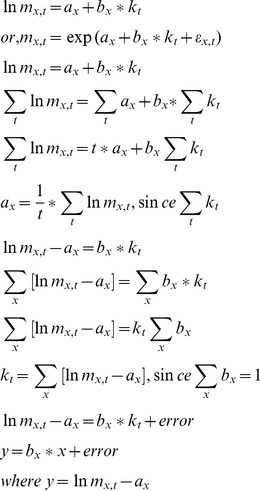



### Construction of life table

To understand the mortality pattern in the future, life table has been constructed using the forecasted age specific death rates. For constructing life table the usual method, the death rates for 0–1, 1–4, and other remaining five years age group are required. But, the SRS provides the death rates in five year age groups starting from 0–4. The _n_m_x_ values are converted into the _n_q_x_ by employing Greville’s formula [21].
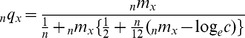



For the age group 0–4, the _n_q_x_ values are calculated using the expression obtained by Roy and Lahiri (1987) [7]. They obtained the relation by establishing a linear regression between _5_p_0_ ( = 1- _5_q_0_) and _5_m_0_ using the relevant data from the South Asian pattern of the united nation model life table for the developing countries. The expression is given as

- For males 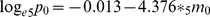

- For females 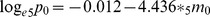

The other life table functions have been calculated in the usual manner. The number of person years lived between the age x and x+n have been obtained by the relation
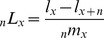



**Table 1:** Estimated values of a_x_ and b_x_ for males and females for India.

Age Group	Males		Females	
	a_x_	b_x_	a_x_	b_x_
0–4	3.18258	0.167421	3.27694	0.10662
5–9	0.75033	0.174153	0.94346	0.12761
10–14	0.23408	0.102773	0.31963	0.07205
15–19	0.50479	0.063267	0.79077	0.06393
20–24	0.79864	0.049129	1.05457	0.06884
25–29	0.94551	0.011702	1.02698	0.06560
30–34	1.16234	0.013489	1.04804	0.06490
35–39	1.42516	0.016473	1.16079	0.06020
40–44	1.74872	0.035294	1.3827	0.06366
45–49	2.13219	0.052644	1.71023	0.05448
50–54	2.55527	0.062898	2.16092	0.06595
55–59	2.95287	0.016848	2.60667	0.04682
60–64	3.39943	0.065659	3.12556	0.05864
65–69	3.80441	0.050237	3.54627	0.04515
70–74/70+	4.45976	0.073013	4.35898	0.04498

**Table 2:** Estimated k_t_ values during 1981–2007 for India.

Year	Males	Females
1981	2.96546	4.50611
1982	1.99323	3.10828
1983	2.41485	3.60462
1984	2.96047	4.35748
1985	2.74139	3.75933
1986	1.90802	2.78695
1987	0.86688	2.19857
1988	1.46479	2.32305
1989	0.87010	1.05966
1990	0.43216	0.81630
1991	1.05558	1.12404
1992	0.59509	1.79171
1993	−0.44236	0.11221
1994	−0.22169	−0.26583
1995	−0.41250	−0.71336
1996	−0.93951	−0.56353
1997	−0.31707	−0.39211
1998	−0.37172	−0.63382
1999	−0.65687	−1.30876
2000	−0.92770	−1.72363
2001	−1.13565	−1.90722
2002	−1.67823	−2.62658
2003	−2.35403	−3.77483
2004	−2.77614	−4.48392
2005	−2.84387	−3.97370
2006	−2.51834	−4.45067
2007	−2.67234	−4.73034

**Table 3:** Observed and expected age specific death rates for India.

Age Group	Males								Females							
	1990		1995		2000		2005		1990		1995		2000		2005	
	Obs	Exp	Obs	Exp	Obs	Exp	Obs	Exp	Obs	Exp	Obs	Exp	Obs	Exp	Obs	Exp
0–4	24.8	25.9	23.2	22.5	18.6	20.6	16.4	15	27.9	28.9	25.3	24.6	20.6	22	18.2	17.3
5–9	2.3	2.5	2.2	1.8	1.5	1.7	1.5	1.4	2.8	3	2.7	2.4	2	2	1.7	1.5
10–14	1.4	1.3	1.3	1.2	1.2	1.1	1.1	1	1.4	1.6	1.4	1.3	1.3	1.1	1.1	1
15–19	1.7	1.6	1.7	1.6	1.6	1.4	1.5	1.7	2.5	2.2	2	2.1	2.2	1.7	1.9	2.2
20–24	2.4	2.2	2.1	2.2	2.2	1.9	2	2.2	3.1	2.8	2.7	2.6	2.7	2.1	2.1	2.9
25–29	2.5	2.6	2.6	2.5	2.9	2.5	2.3	2.6	2.8	2.7	2.6	2.5	2.7	2.2	2.2	2.8
30–34	3.1	3.2	3.1	3.1	3.3	3.1	3	3.2	2.9	2.7	2.7	2.5	2.8	2.1	2	2.9
35–39	3.9	4.1	3.7	4	4.4	4	3.9	4.2	3.2	3.1	3.1	2.7	3	2.4	2.4	3.2
40–44	5.7	5.7	5.5	5.3	5.5	5.7	5	5.7	4.4	3.8	3.8	3.1	3.3	4	3	4
45–49	9	8.1	8.1	7.3	8.1	8.4	7	8.4	6.3	5.1	5.2	4.3	5.3	5.5	4.4	5.5
50–54	13.2	12.1	12	10.8	12.2	12.9	10.8	12.9	9	7.7	8.5	6.7	7.4	8.7	6.4	8.7
55–59	20.9	18.8	17.5	18.4	19.1	19.2	15.4	19.2	14.4	12.4	11.8	11	13.4	13.6	10.9	13.6
60–64	28.9	26.8	28	25.1	26.6	29.9	24.2	29.9	23	19.5	21.5	17.3	19.7	22.8	18.2	22.8
65–69	47.3	39.9	41.6	44.9	44.2	44.9	37.7	44.9	37.8	29.2	30.5	34.7	32.3	34.7	27.7	34.7
70–74/70+	81.4	70.6	97.2	86.5	73.8	86.5	76.7	86.5	79.4	73.9	81	68.2	64.1	78.2	68.2	78.2

***Note:*** Obs – Observed.

Exp - Expected.

## Results and Discussions

**Table 1** provides the estimated values of age specific constants a_x_ and b_x,_ where a_x_ coefficient describes the average shape of the age schedule of mortality and coefficient b_x_ shows the speed of decline of the _n_m_x_ values. The a_x_ values can be considered as approximate indicator of level of mortality decline. The a_x_ values are constant for the particular age group. Starting from the lowest age group, the a_x_ values continues to decline upto 25–29 and then increased upto maximum value of 4.45 for the age group 70+. For females also the a_x_ decreases upto the age-group (20–24) and then increases thereafter and reach to a maximum value of 4.35 for last age group. In comparison to male a_x_ values, the female a_x_ values are found to be higher for lower age groups 5–9 to 25–29, indicating high level of female mortality in these age groups. The coefficient b_x_ shows the speed of decline of the _n_m_x_ values. It is clear from the table that the death rates in the age group 0–4 and 5–9 decline much faster than other age groups. The decline in the coefficient b_x_ for females is found to be less in comparison to males in the age group 0–4, 5–9, and 10–14. Whereas the speed of decline for females is higher from the age groups 15–19 till the age 55–59. This may be accredited to improvement in maternal mortality due to advancement in health facility and special care given to women.

**Table 4:** Forecasts of mortality index k from (0, 1, 0) model, India.

Year	Male			Female		
	k_t_	LCL	UCL	k_t_	LCL	UCL
**2008**	−2.89	−3.97	−1.81	−5.09	−6.43	−3.75
**2009**	−3.11	−4.66	−1.55	−5.44	−7.37	−3.51
**2010**	−3.32	−5.26	−1.38	−5.80	−8.20	−3.39
**2011**	−3.54	−5.82	−1.26	−6.15	−8.98	−3.33
**2012**	−3.76	−6.34	−1.17	−6.51	−9.72	−3.30
**2013**	−3.97	−6.85	−1.09	−6.86	−10.43	−3.29
**2014**	−4.19	−7.35	−1.03	−7.22	−11.14	−3.30
**2015**	−4.41	−7.83	−0.98	−7.57	−11.82	−3.32
**2016**	−4.62	−8.31	−0.94	−7.93	−12.50	−3.35
**2017**	−4.84	−8.78	−0.90	−8.28	−13.17	−3.39
**2018**	−5.06	−9.25	−0.86	−8.64	−13.84	−3.44
**2019**	−5.27	−9.71	−0.84	−8.99	−14.50	−3.49
**2020**	−5.49	−10.17	−0.81	−9.35	−15.15	−3.54
**2021**	−5.71	−10.63	−0.79	−9.70	−15.81	−3.60
**2022**	−5.92	−11.08	−0.77	−10.06	−16.45	−3.67
**2023**	−6.14	−11.53	−0.75	−10.41	−17.10	−3.73
**2024**	−6.36	−11.98	−0.74	−10.77	−17.74	−3.80
**2025**	−6.58	−12.42	−0.73	−11.12	−18.38	−3.87

**Note:** k_t_ values at 95% confidence intervals.

LCL: Lower Confidence Limit.

UCL: Upper Confidence Limit.

**Table 2** gives the estimated values of k_t_ (index of the level of mortality) separately for males and females. It is clear that index of level of mortality has gone down over the year for both sexes. The decline in the index of level of mortality is more prominent in the case of females than males.

**Table 5:** Forecasts of age specific death rates for India.

Age Group	Age specific death rates(male)				Age specific death rates(female)			
	2010	2015	2020	2025	2010	2015	2020	2025
**0–4**	13.82	11.53	9.61	8.02	14.28	11.82	9.78	8.09
**5–9**	1.19	0.98	0.81	0.67	1.23	0.98	0.78	0.62
**10–14**	0.90	0.80	0.72	0.64	0.91	0.80	0.70	0.62
**15–19**	1.34	1.25	1.17	1.09	1.52	1.36	1.21	1.08
**20–24**	1.89	1.79	1.70	1.61	1.93	1.70	1.51	1.33
**25–29**	2.48	2.44	2.41	2.38	1.91	1.70	1.51	1.35
**30–34**	3.06	3.01	2.97	2.93	1.96	1.74	1.55	1.39
**35–39**	3.94	3.87	3.80	3.73	2.25	2.02	1.82	1.63
**40–44**	5.11	4.92	4.73	4.56	2.76	2.46	2.20	1.96
**45–49**	7.08	6.69	6.32	5.97	4.03	3.66	3.32	3.02
**50–54**	10.45	9.76	9.11	8.51	5.92	5.27	4.68	4.17
**55–59**	18.12	17.79	17.47	17.15	10.33	9.51	8.75	8.05
**60–64**	24.08	22.42	20.88	19.45	16.21	14.61	13.16	11.86
**65–69**	38.00	35.98	34.07	32.27	26.70	24.64	22.74	20.99
**70–74/70+**	67.84	62.68	57.91	53.50	60.24	55.61	51.34	47.40

**Table 3** presents the observed (SRS) and expected (calculated) age specific death rates for males and females. After estimating the parameter a_x_, b_x_ and k_t_, the age specific death rates are estimated for four periods namely 1990, 1995, 2000 and 2005. It can be seen that there is not much difference in the expected and observed values but there is slight difference in the last age group. One important fact that has been done in the table is that, an adjustment has been felt necessary given the problem of the age break up. Prior to 1995, age specific break up is upto 70+ but the subsequent years the break up has been increase to 85+, which result in minor difference in the last age group. It indicates the suitability of the model for India. The Graphical presentation of age specific death rates (both observed and expected) are shown in the figure and for the most of the age groups the fit seems to be reasonably good.

**Table 6:** Forecasts of numbers surviving to exact ages for selected years, India.

Age Group	l_x_(male)				l_x_(female)			
	2010	2015	2020	2025	2010	2015	2020	2025
**0–4**	100000	100000	100000	100000.00	100000	100000	100000	100000
**5–9**	92916	93852	94643	95304	92742	93760	94612	95324
**10–14**	92364	93393	94261	94985	92173	93301	94244	95029
**15–19**	91950	93020	93922	94682	91755	92929	93915	94735
**20–24**	91336	92440	93374	94167	91060	92299	93348	94225
**25–29**	90476	91616	92584	93412	90185	91518	92646	93600
**30–34**	89361	90505	91474	92307	89328	90743	91949	92970
**35–39**	88003	89152	90125	90963	88456	89956	91239	92326
**40–44**	86285	87442	88428	89281	87466	89052	90412	91576
**45–49**	84106	85316	86359	87267	86267	87963	89422	90683
**50–54**	81177	82506	83670	84698	84545	86367	87949	89323
**55–59**	77037	78569	79938	81164	82076	84119	85913	87479
**60–64**	70342	71861	73230	74474	77937	80205	82230	84022
**65–69**	62329	64210	65944	67548	71852	74540	76981	79174
**70–74/70+**	51473	53572	55554	57426	62830	65862	68674	71257

The forecasted values of k_t_ are given in the **table 4**. The 95 percent confidence interval is also given in the same table. The forecasted values are given up to the year 2025. It is clear from the table that the level of mortality will decline for both sexes, but the decline in the level of mortality is likely to be higher in the case of females in comparison to males. The age specific death rates have been forecasted using the estimated values of a_x_, b_x_ and forecasted values of k_t_ using autoregressive integrated moving average model.

**Table 7:** Comparative forecasted life expectancy at birth between Lee-Carter model and Registrar General of India by sex, India.

Year	Forecast of life expectancies at birth by sexusing Lee Carter model for India		Life expectancies at birth for India as projected by Registrar General of India	
	Male	Female	Male	Female
**2010**	66.62	71.1	65.8	68.1
**2015**	68.63	73.81	67.3	69.6
**2020**	70.65	76.52	68.8	71.1
**2025**	72.71	79.27	69.8	72.3

**Table 5** reveals the forecasted age specific death rates for the year 2010, 2015, 2020 and 2025 for both sexes. The age specific death rates have been forecasted using the estimated values of a_x_, b_x_ and forecasted values of k_t_ using autoregressive integrated moving average model. The death rates in India as a whole are expected to decline rapidly. Under five mortality is expected to decline to 8 per thousand for both the sexes by the year 2025.Overall the death rates for the age group 5–9 and 10–14 are expected to go below one per thousand by the year 2025 for both the sexes. The age specific death rates are lower for females as compared to males in all the age groups except 0–4 age group.

**Figure 2:**
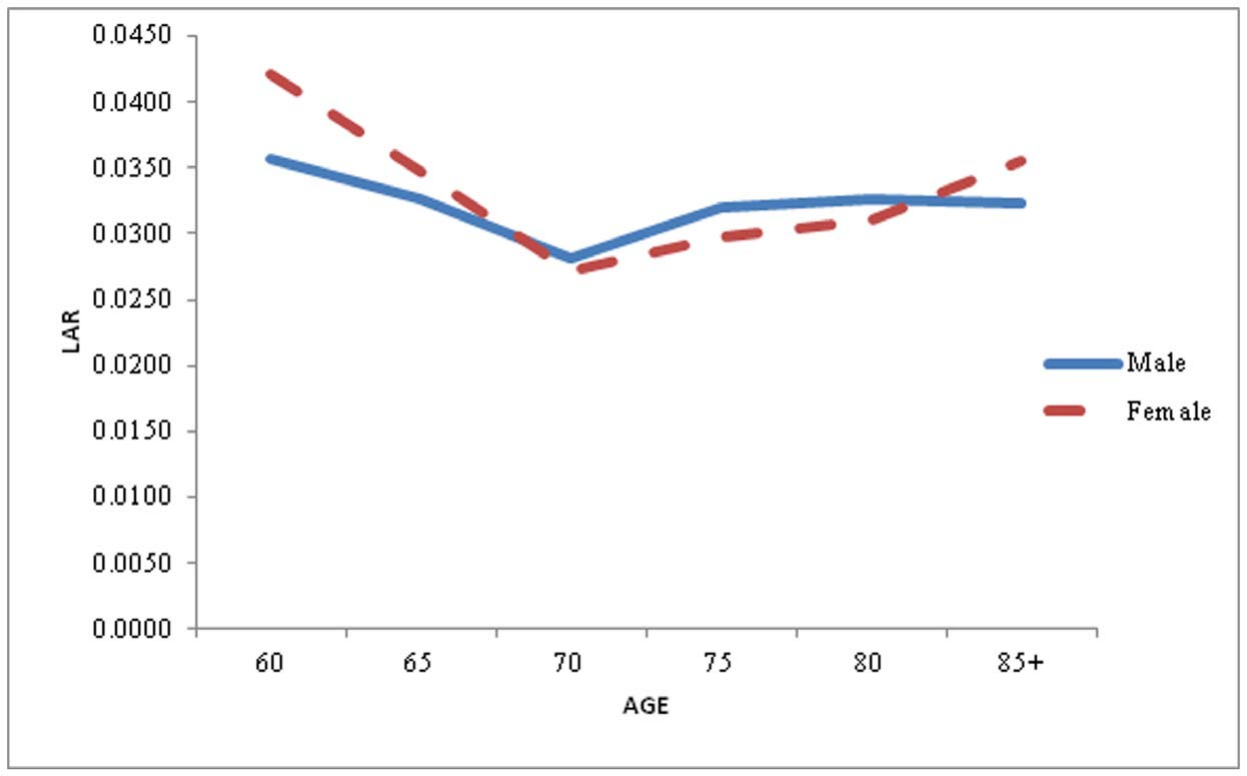
Life Table Aging Rate (LAR) for Male and Female in the older age, India, 1993.

**Table 6** present the number of serving to exact age for both sexes. The l_x_ values have been constructed for the years 2010, 2015, 2020 and 2025. In 2010, about 51 percent of the male births and 62 percent of the female birth are expected to survive up to age 70. By 2025, this percentage is forecasted to increase to 57 percent and 71 percent respectively. About 95 percent of the total live births are forecasted to survive to age five by the year 2025.

Life expectancy at birth is important function of a life table**.** **Table 7** present the forecasted life expectancies at birth by using Lee-Carter and life expectancies at birth for India as projected by Registrar General of India. The e_0_°value for India is forecasted to be 72.71 years for males and 79.27 for females by the year 2025.Comprison with the level of e_0_° given by the Registrar General of India reveal that the e_0_° given by Lee-carter model is higher than that of Registrar General of India.

**Figure 3:**
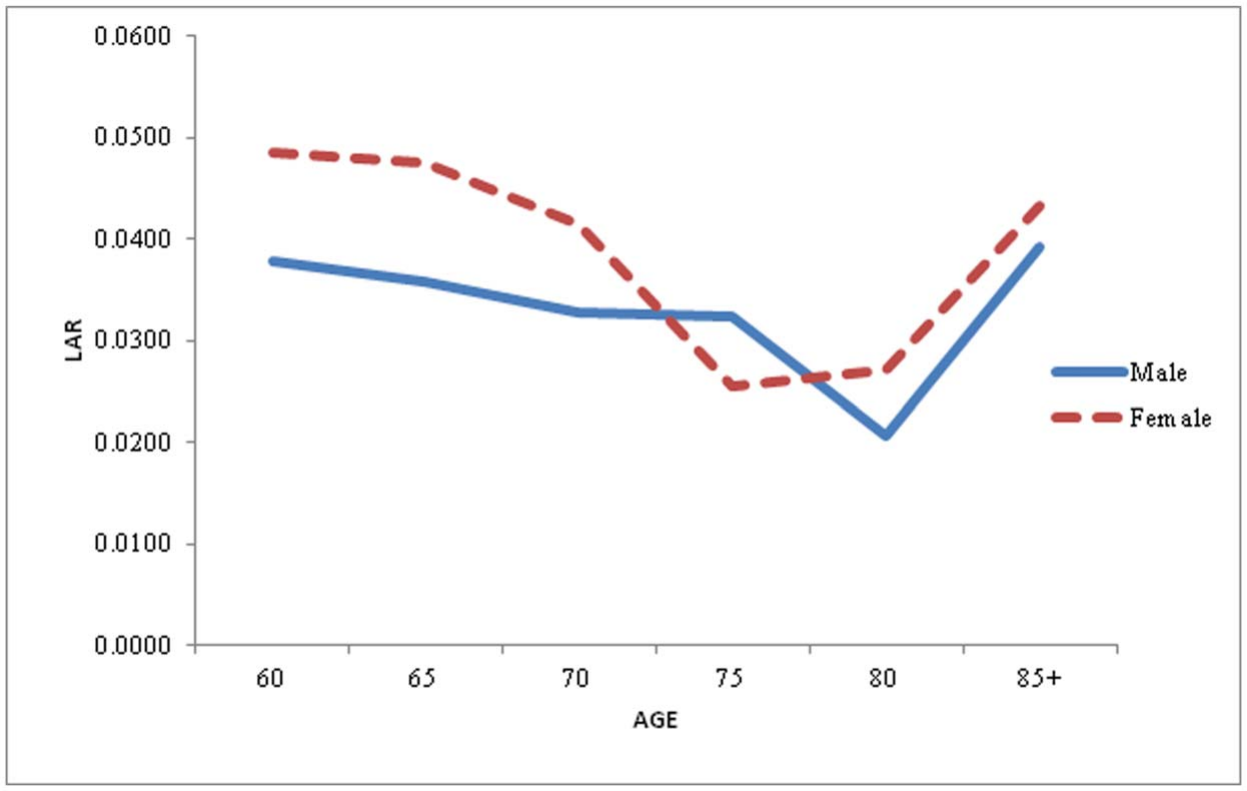
Life Table Aging Rate (LAR) for Male and Female in the older age, India, 1999.

The Life Table Aging Rate (LAR) has been calculated for India separately for male and female. For understanding of mortality decline and changes, last 15 years data has been considered i.e.1993–2007. LAR has been calculated for older age, generally, age 60 and above for male and female as mentioned methodology. **Figure 2** gives the rate of mortality change (may be accelerating or decelerating) for male and female, 1993. Overall the result seems compatible, it has been observed that LAR is decelerating, up to age 70 and after that it slightly increase for the oldest age. This means that mortality has been decreasing upto age 70 and later on mortality is increasing with constant rate i.e. mortality is accelerating for age 75 and above. However, it has been clearly seen that the pace of mortality decline is more pronounced for female against male counterpart. Also the age pattern for mortality acceleration for male remain slightly higher than female for age 80, beyond that its value become stagnant.

**Figure 4:**
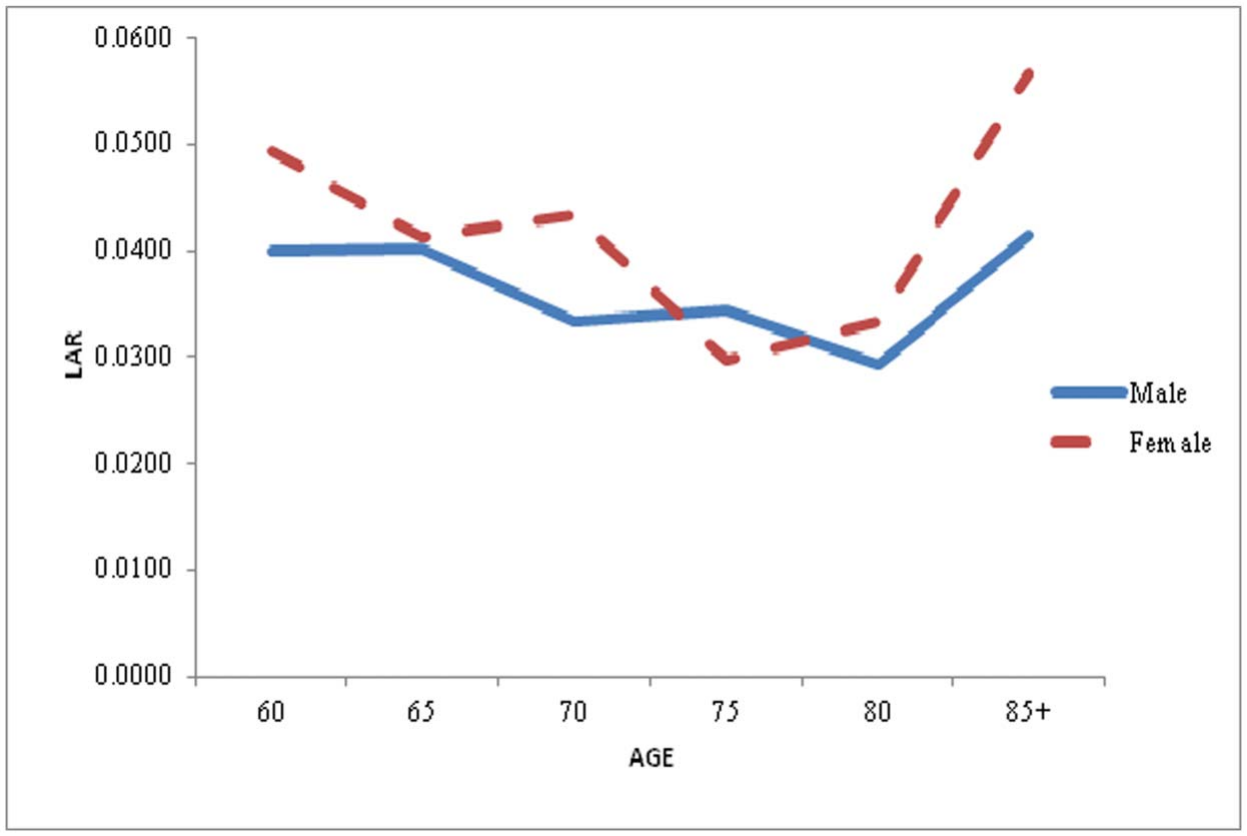
Life Table Aging Rate (LAR) for Male and Female in the older age, India, 2006.

Again LAR has been calculated separately for male and female for the year 1999. Also from **figure 3**, it has been observed that age related mortality continue to decrease i.e. mortality is decelerating although LAR decline is more prominent in male as compared to female upto age 70. However, for female in age 75, there is decrease in mortality as compared to male after that LAR for female has been increasing for oldest age. It is clearly visible from the figure that mortality is accelerating with constant rate beyond age 80. And this is true for both male and female.

For the better understanding of pattern and prospects of mortality acceleration and deceleration, Again LAR has been calculated separately for male and female for the year 2006. **Figure 4** gives the same pattern has been seeing here what the previous two graph shows. Here also Mortality is decelerating, for both male and female, upto age 70 after that mortality is accelerating for age 75 and above. But the pace of mortality decline is more prominent in male compared to female. For instance in case male the LAR for age 70 is 0.0333 means that death rate rising at an exponential rate of 3.33 percent where as for female, LAR for same age is 0.0433 means that death rate is rising at an exponential rate of 4.33. Here the rising of mortality is more in female as compared to male. The only exception is for age 75 where LAR is less for female as compared to male.

The above discussion clearly pointed out that mortality rate is declining with constant rate upto age 70 after that mortality is accelerating beyond age 70 and this holds true for both sexes i.e. male and female. The rates of mortality increase at oldest of old group because this age group is more vulnerable to chronic disease and epidemiological studies have shown that there are identifiable risk factors for virtually every disease, marked by senility. Also, the risk factor vary across the populations, some persons are more vulnerable to the disease than others. It follows that if the overall adult mortality declines, then the pattern of mortality deceleration should shift to older ages. This relationship between the mortality level and the mortality deceleration was dominated in an earlier simulation study [22].

Why does the deceleration occur? Although an apparent slowdown can be attributed to inaccurate data for some human populations [23], [24], the trend has been observed using accurate data as well. There at least two possible explanations for this phenomenon: the heterogeneity hypothesis and the individual -risk hypothesis [25].

According to the heterogeneity hypothesis, the deceleration is a statistical effect of selection through the attrition of mortality. Because the frailer tend to die at younger ages, survivors to older age tend to have more favorable health endowments and/or healthy lifestyles. This argument has been supported by several studies based on mathematical models [26], [27] and simulations [28]–[30]. Some parametric models on relationship between physiological changes and mortality pattern suggest that selection survival should cause decelerations of age-related increases in both mortality and disability at very old ages [31], [32].

According to the individual-risk hypothesis, the age related increase of mortality risk for individuals slow down at older ages for one or more reasons. There are three versions of the individual-risk hypothesis: physiological, evolutionary and reliability-theoretical. First, if the “rate of living” is slower in older age, so may be the “rate of aging”. For example, there is much direct and indirect evidence of negative correlations, both between and within species, between rate-of-living measures and life expectancy [33], [34]. Furthermore, declines in the rate of living at old ages have been observed for a number of physiological functions [35], [36], including fundamental processes such as cell division [37]. In addition, the development of some disease is slower at older ages [38]–[40].

### Conclusions

In general, the results of the study appear broadly consistent with the heterogeneity hypothesis. It is possible, however, that they are also compatible with specific versions of the individual risk hypothesis. Finally, a shift in the pattern of deceleration to older ages might have been caused by a delay of senescent processes, brought about by improvement in the health of the elderly during the recent decades [32].

Life expectancy is increasing linearly overtime at the rate of 0.45 per year, which is fluctuating but periodic in nature. Partially the periodic nature is attributed to the level of heterogeneity and the extent of vulnerability of the group. For all younger and older age group, the trend is almost linear however, for 0–4 age group the trend is steeper. It is seen that the life expectancy for females is considerably higher as compared to males. It is clearly pointed out that level of mortality is not declining but the mortality rate is decelerating and is further expected to decelerate in future. The process of deceleration is clearly visible in older ages. Our estimates are higher than SRS estimates which is further underpinned by deceleration of mortality acceleration in older age group.

The estimates revealed that in past decades the mortality rates for females is higher compared to males in adult age group. For children and older age group, mortality rate for males were higher than for females. In contrast, in projected years, the decline in the death rates for the age groups 0–4 and 5–9 are much faster than those in the higher age groups and the decline for females is higher than those of males in the reproductive ages which may be due to decline in maternal mortality and utilization of health care services.

Life tables were constructed using the forecasted death rates from the Lee-Carter model. By the year 2025, the death rates for age groups 5–9 and 10–14 will go below one per thousand. Analysis revealed that, 71 percentage of the total female birth and 57 percentage of total male birth will survive up to age 70 and above. The life expectancy at birth obtained from Lee-Carter model is higher than from those obtained from the estimates provided by Registrar General of India. The life expectancy at birth for females is higher than males by 6 years, signifying the increasing gap between both sexes.

The above discussion ascertained that mortality rate is decelerating with constant rate upto age 70, and thereafter, the rate is accelerating for oldest of old. This holds true for both sexes i.e. male and female. The rise in mortality rates in oldest of old group is attributable to chronic disease and epidemiological studies have ascertained that there are identifiable risk factors for virtually every disease, marked by senility in the older age group.

This paper explores some policy implication of declining mortality among the elderly population. There are two views of how health program will affect the social burden of caring for the aged. One holds that prolonging the lives of frail individuals will result in rapidly increasing medical and other costs per aged person. A second view suggests that health progress and behavioral changes will reduce both mortality and morbidity rates, lowering the average cost per person of caring for the aged.
